# Analysis of lumbar spine loading during walking in patients with chronic low back pain and healthy controls: An OpenSim-Based study

**DOI:** 10.3389/fbioe.2024.1377767

**Published:** 2024-05-13

**Authors:** Zhuodong Zhang, Jihua Zou, Pengcheng Lu, Jinjing Hu, Yuxin Cai, Chongwu Xiao, Gege Li, Qing Zeng, Manxu Zheng, GuoZhi Huang

**Affiliations:** ^1^ Department of Rehabilitation Medicine, Zhujiang Hospital, Southern Medical University, Guangzhou, China; ^2^ School of Rehabilitation Medicine, Southern Medical University, Guangzhou, China; ^3^ Department of Rehabilitation Studies, The Hong Kong Polytechnic University, Hong Kong, Hong Kong SAR, China

**Keywords:** low back pain, the multibody model, lumbar spine load, OpenSim, walking

## Abstract

Low back pain (LBP) is one of the most prevalent and disabling disease worldwide. However, the specific biomechanical changes due to LBP are still controversial. The purpose of this study was to estimate the lumbar and lower limb kinematics, lumbar moments and loads, muscle forces and activation during walking in healthy adults and LBP. A total of 18 healthy controls and 19 patients with chronic LBP were tested for walking at a comfortable speed. The kinematic and dynamic data of the subjects were collected by 3D motion capture system and force plates respectively, and then the motion simulation was performed by OpenSim. The OpenSim musculoskeletal model was used to calculate lumbar, hip, knee and ankle joint angle variations, lumbar moments and loads, muscle forces and activation of eight major lumbar muscles. In our results, significant lower lumbar axial rotation angle, lumbar flexion/extension and axial rotation moments, as well as the muscle forces of the four muscles and muscle activation of two muscles were found in patients with LBP than those of the healthy controls (*p* < 0.05). This study may help providing theoretical support for the evaluation and rehabilitation treatment intervention of patients with LBP.

## 1 Introduction

Low back pain (LBP) is the pain, muscle tension, or stiffness located below the costal margin and above the subgluteal fold, with or without leg pain, when LBP exceeded 3 months (Fourney et al., 2011), it is defined as chronic LBP. The mean prevalence of LBP in adults is approximately 12%, and the lifetime prevalence is approximately 40% (Hoy et al., 2012). LBP is the most prevalent and disabling disease considered for rehabilitation worldwide (Cieza et al., 2021). Despite its widespread occurrence, the underlying mechanisms of LBP are not well understood, largely due to insufficient assessment of biomechanical factors (O'Sullivan, 2005).

Chronic LBP is often aggravated by changes in spinal biomechanics. In lumbar biomechanical analysis, compression, shear, and twisting forces on the intervertebral discs are key indicators of discogenic LBP caused by degenerative changes in the lumbar spine (Marras et al., 2001; Adams, 2004). Excessive loads can cause annular fissures, disc herniation, and other degenerative changes, resulting in chronic LBP (Creighton et al., 2023). This emphasizes the importance of accurately assessing the differences in lumbar load between individuals with and without LBP. Repeated exposure to high-intensity forces may damage spinal integrity, especially during everyday activities such as walking (Gombatto et al., 2015; Christe et al., 2016). In the current field of biomechanical research on LBP, the focus is primarily limited to specific lumbar segments. However, this method overlooks the impact of movements in other parts of the body on the lumbar spine, lacking a holistic analysis of motion. We need to understand more comprehensively the effects of body movements on the lumbar spine and validate these theories through experiments. This will help us gain a deeper understanding of the biomechanical mechanisms of LBP, providing more effective strategies for the prevention and treatment of LBP (Owen et al., 2020; Pocovi et al., 2022).

To elucidate the biomechanical intricacies of LBP, traditional methodologies have employed cadaveric experiments (Nachemson, 1981)or the implantation of pressure sensors *in vivo* (Sato et al., 1999)to quantify lumbar load during routine activities. However, these approaches present limitations: cadaver studies fail to mimic the dynamic physiological responses inherent in living tissues (Von Forell et al., 2015), and implanted sensors, while invasive, fall short in replicating the natural load conditions encountered by an active human body, rendering them unsuitable for monitoring the daily activities of individuals with or without LBP (Ferrara et al., 2005). Therefore, biomechanical models of different complexities have become key tools in this research field, providing a non-invasive approach for comprehensive analysis of the mechanical basis of the human neuromuscular system. The main methods include finite element models and multibody models (Roupa et al., 2022).

The finite element method (FEM) is a computational technique that discretizes a continuous model into a finite number of non-overlapping elements in space and time (Castro et al., 2015). Kim et al. (Kim et al., 1991)initially utilized a nonlinear three-dimensional FEM to investigate the vertical compressive forces exerted on the lumbar intervertebral disc. Later, Simon (Simon et al., 1985), Lee (Lee et al., 2000), and Williams (Williams JR et al., 2004) further refined the research related to the lumbar FEM. However, the FEM often simplify the geometric shape and constitutive relationship of the lumbar spine. Moreover, these models are unable to simulate the kinematics of the lumbar spine and muscle activation during holistic movement. Thus FEM is not suitable for this study.

The multibody model is a biomechanical motion analysis model that decomposes the system into a set of rigid bodies connected by joints, which can be used to study the impact of overall motion analysis on a specific joint (Ma et al., 2023). Cappozzo (Cappozzo, 1984), Callaghan et al. (Callaghan et al., 1999)and Kuai et al. (2017a) used different lumbar multibody models to simulate and predict the lumbar spine load under different movements. Although, the construction of the multibody model does not take into account the inter-individual variability, it currently stands as the only effective method to simultaneously obtain *in vivo* kinematics, lumbar loading, muscle force and activation. Previous multibody lumbar studies only focused on limited lumbar segments, such as the lumbar L3-L4 and L4-L5 segments, ignoring the impact of overall body mass and overall movement on lumbar biomechanics. These limitations hinder comprehensive investigations into potential biomechanical differences and underlying mechanisms, potentially leading to unsupported conclusions.

OpenSim is an open source software developed by Stanford University for the study and design of biomechanical and neuro-controlled movements (Delp et al., 2007). The software visualizes complex biomechanical analysis and simulation, providing a powerful tool for understanding human motion mechanisms and designing solutions. The researchers used recent experimental studies to improve previously published models and continue to add new models to expand OpenSim’s possible research applications (Seth et al., 2018). Models of muscle mechanics have typically been validated against experimental data obtained from animals (Millard et al., 2013). Other simulation results were verified by each model developer. The latest advancements in the full-body lumbar spine (FBLS) model (Raabe and Chaudhari, 2016)provide a comprehensive musculoskeletal modeling approach. It considers the impacts and interactions of the whole body, enabling precise load calculations for each lumbar segment. This model was validated against measured EMG, joint angle and moments (Novacheck, 1998; Brown et al., 2014)and previous simulation results (Hamner et al., 2010). But the FBLS model also has some limitations, the model contains 324 musculotendon actuators, the computational cost to create simulations with this model is higher than simpler models. In addition, the model can not be able to perform computed muscle control (CMC) or forward dynamics. It can only simulate muscle force and activation through static optimization (Lin et al., 2011), which may affect the accuracy of the results to some extent. This model has been applied to biomechanical studies of running (Raabe and Chaudhari, 2018) and crawling (Li et al., 2020). However, there is still no consensus on whether there are significant differences in kinematics and dynamics between LBP patients and healthy individuals, and there is a lack of personalized data for LBP patients. The purpose of this study was to estimate the lumbar and lower limb kinematics, lumbar moments and loads, and muscle activation during walking in healthy adults and LBP.

## 2 Methods

### 2.1 Participants

This study recruited a total of 37 participants from the community and online, including 18 healthy individuals and 19 participants with chronic LBP. Only one subject with LBP had left side pain, while the others had right side pain. Inclusion criteria followed the non-specific LBP diagnosis guidelines of the American College of Physicians and the American Pain Society (Chou et al., 2007). The chronic LBP patients who met the following criteria were included in the study: (1) clinical diagnosis of non-specific LBP or discomfort for >3 months, with a Visual Analog Scale (VAS) (Chiarotto et al., 2019)score was greater than 30mm; (2) age 18–75 years. A healthy control group meeting the following criteria was included in the study: (1) no incidence of low back pain in 2 years; (2) age 18–75 years. The key exclusion criteria were as follows: (1) pregnancy; (2) a history of waist trauma or waist/abdominal surgery in the past 2 years; (3) a history of nerve roots symptoms, spine fracture, infection, lumbar malignancy, or LBP caused by any other disease; and (4) patients suffering from hypertension, heart disease, Parkinson’s disease, and other conditions that were not suitable for intense exercise; (5) inability to walk independently or an abnormal gait. Patients with LBP and healthy controls were matched for age and gender. The mean age of the LBP group was 23.95 years, and the average age of the healthy group was 23.44 years (Table 1). All participants had no recent history of back injury or surgery within the last 2 years. The individuals with a clinical diagnosis of nonspecific chronic LBP (Chou et al., 2007)that persists for more than 3 months, and their Visual Analog Scale (VAS) (Chiarotto et al., 2019)score was greater than 30 mm. The Ethics Committee of Zhujiang Hospital of Southern Medical University approved this study (2023-KY-017). Informed consent was obtained from all participants prior to the experiment.

**TABLE 1:** Baseline characteristics (X (sd)).

Characteristic	Low back pain group (n = 19)	Healthy control group (n = 18)	*P*
Age (years)	23.9 (1.5)	23.4 (1.6)	0.335
Gender			0.873
Male	9 (47%)	9 (50%)	
Female	10 (53%)	9 (50%)	
BMI (kg/m2)	21.7 (2.8)	22.3 (3.7)	0.586
Timed up-and-go (s)	10.6 (1.1)	8.2 (0.6)	**<0.001**
Gait cycle (s)	1.4 (0.1)	1.3 (0.1)	0.856
rest thickness of LTrA (mm)	3.5 (1.0)	3.2 (0.6)	0.327
rest thickness of RTrA (mm)	3.2 (0.7)	3.2 (0.6)	0.946
rest thickness of LMF (mm)	26.7 (4.3)	29.3 (5.2)	0.106
rest thickness of RMF (mm)	26.4 (3.6)	28.4 (4.6)	0.142
contracted thickness of LTrA (mm)	4.7 (1.1)	5.1 (1.0)	0.249
contracted thickness of RTrA (mm)	4.6 (1.0)	5.2 (0.9)	0.123
contracted thickness of LMF (mm)	35.1 (4.1)	39.0 (5.5)	**0.022**
contracted thickness of RMF (mm)	35.1 (4.2)	38.9 (5.8)	**0.033**
contraction rate of LTrA (%)	43.1 (17.0)	61.0 (15.9)	**0.002**
contraction rate of RTrA (%)	46.3 (19.7)	60.5 (16.1)	**0.022**
contraction rate of RMF (%)	33.1 (13.0)	34.2 (9.8)	0.784
contraction rate of LMF (%)	33.9 (11.8)	37.2 (8.1)	0.321

Abbreviations: BMI, body mass index; LTrA, left transverse abdominal muscle; RTrA, right transverse abdominal muscle; LMF, left multifidus muscle; RMF, right multifidus muscle.

The bold values indicate *P* value ≤0.05.

### 2.2 Experimental procedures

Before starting the test, the subjects were instructed to wear tight-fitting clothing and were informed about how to perform the walking tests within the designated area. When the test started, participants were first asked to stand in the test area in an anatomical position (standing upright, facing forward, eyes looking straight ahead, feet together, toes pointing forward, both upper limbs hanging at the sides of the torso with palms facing forward) (Hansen and Netter, 2009) to collect the necessary static data for the test. Subjects were then instructed to walk through the force plates at their own comfortable pace. After a single pass through the force plates, they were required to turn and walk back to the starting point at a comfortable pace, repeating this process back-and-forth five times. A successful trail was defined as having both feet on two force plates during a gait cycle.

### 2.3 Data collection

**FIGURE 1:**
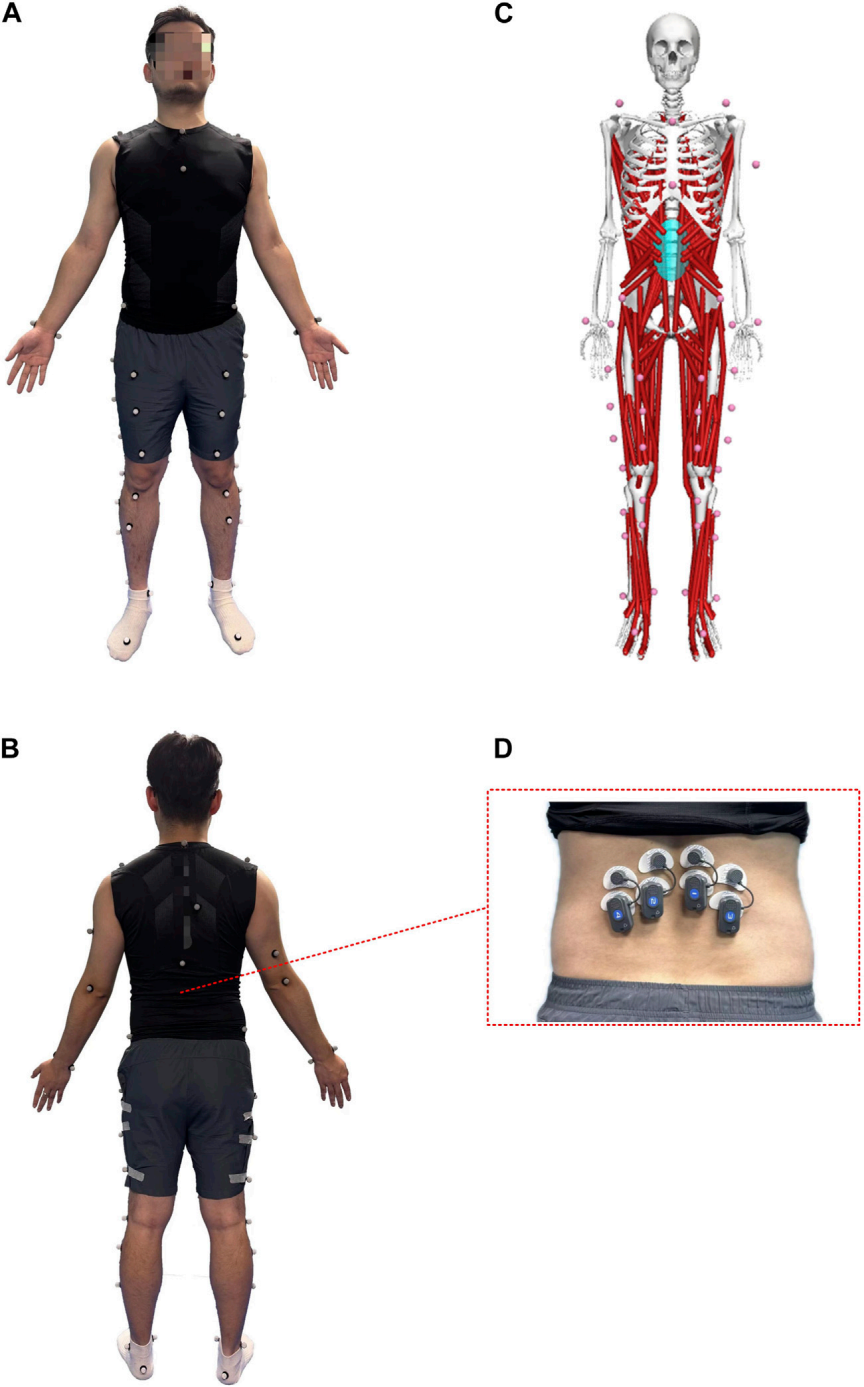
Anterior **(A)** and posterior **(B)** views of the lower body Point-Cluster marker set with the upper body Plug-In Gait marker set; **(C)** OpenSim musculoskeletal model; **(D)** The EMG electrode shows the iliocostalis muscle.

The kinematic data were recorded with a 6-camera infrared 3D motion capture system (BTS SMART-DX EVO2) with an acquisition frequency of 100 Hz. A total of 49 Reflective Markers (14.0 mm diameter) were affixed to the subjects’ whole body (Figures 1A,B) (Jamison et al., 2013). The results of the static and dynamic tests showed an error lower than 0.1 mm within a volume of 4 m × 3 m × 3 m (L × W × H), similar to other commercial systems usually used in biomechanics. Ground reaction forces were collected using 2 force plates (BTS P6000) at 1,000 Hz. Surface electromyography (EMG) data of the left and right erector spinae muscles (BTS FREEEMG 300) at 1,000 Hz were placed according to SENIAM guidelines (Figure 1D) (Stegeman and Hermens, 2007). The data of 3D kinematic and GRF data, as well as Surface EMG data, were updated and adjusted in the BTS system to ensure temporal uniformity of data acquisition. The muscle thickness of Musculi transversus abdominis (TrA) and Multifidus muscle (MF) was measured using a Terason uSmart 3,300 ultrasound (Terason, Burlington, United States).

The data collection process commences with the initial step of gathering static standing data. Following this, participants were instructed to walk at their comfortable pace during the gait tests, with marker trajectory data, ground reaction forces data, and surface EMG data being collected simultaneously. Each participant underwent at least five trials to ensure complete gait data collection. A qualified dataset required separate recordings of left and right feet on two different force plates. If data from both feet were recorded on the same force plate, an additional trial was performed. Meanwhile, all collected data were processed by normalization. For TrA thickness, patients were instructed to lie in the supine position with hips flexed to approximately 135° and knees flexed to 90°. They were then asked to take a deep breath, exhale fully, and keep their abdomen relaxed for 5 s. A linear probe was used to measure the thickness above the iliac crest at the axillary front. Muscle thickness was recorded as the distance between the hyperechoic myofascial. For MF thickness, patients were instructed to lie in the prone position with a thin pillow under their abdomen to straighten the lumbar spine. A curve probe was used to measure the thickness at the fourth lumbar spine. Muscle thickness was recorded as the distance from the tip of the articular process to the lower end of subcutaneous fat. The measurement was performed on both left and right sides three times, and the average was used for data analysis (Skeie et al., 2015).

### 2.4 Musculoskeletal modeling and simulation

From the collected data, a complete gait data was selected and input into OpenSim software (version 4.1) for kinematicand dynamic calculations using the Full Body Lumbar Spine (FBLS) model created by Raabe and Chaudhari (Raabe and Chaudhari, 2016). The steps are as follows:

The process began by scaling the original model to match the participant’s height, weight, and body proportions. This was based on the static data collected from the subject, ensuring that the segment ratios of the body align with those of the corresponding subject in the original experiment. Following this, the Inverse Kinematics tool in OpenSim was employed to input the experimentally acquired kinematic data into the model, which then drove the model to obtain the kinematics data during gait. Subsequently, the Residual Reduction Algorithm was applied to optimize the model’s motion data and body segment mass properties. This resulted in an adjusted model and a new set of Inverse Kinematics and Inverse Dynamics outcomes. The next step was to proceed with Static Optimization, which involved allocating net joint moments obtained from Inverse Dynamics to individual muscle fibers frame by frame. This yielded muscle forces and activation levels throughout the motion. Finally, the Joint Reaction Analysis tool was used to calculate the internal loads experienced on the lumbar joints during gait. This allowed us to obtain the compression and shear forces and twisting forces on the L3-S1 lumbar intervertebral disc.

### 2.5 Statistical analysis

Statistical analyses were performed using IBM SPSS v25. The Chi-square test was used for the categorical variable (gender). For continuous variables, the Shapiro–Wilk test was used to test whether the data were normally distributed, and the Levene test was used to test whether the two sets of data were homogeneous. The independent-samples t-test was used for the outcomes (age, years of education, BMI, gait cycle, muscle thickness, axial rotation peak moment, lumbar intervertebral peak load) with a normal distribution and consistent variance in both groups, and the independent-samples Mann-Whitney U test was used for the outcomes (flexion-extension and lateral bending peak moment, muscle peak force) with non-normal distribution or irregular variance. BW normalization was performed on the simulated moment and lumbar Intervertebral load for each subject. All outcomes of continuous variables were described as mean ± standard deviation, and all statistical inferences were performed using two-sided tests, α = 0.05.

## 3 Results

### 3.1 Subjects

There were no significant differences in gender, age, BMI, years of education, or gait cycle between the two groups (Table 1, *p* > 0.05). The TUG test duration, contracted thickness of MF, and contraction rate of TrA significantly differ between the group with LBP and the healthy group (Table 1, *p* < 0.05).

### 3.2 Kinematics

The average gait cycle in the LBP group (1.35 ± 0.10s) was slightly longer than that in the healthy group (1.34 ± 0.13s), but the difference was not significant (Table 1, *p* > 0.05).

**FIGURE 2:**
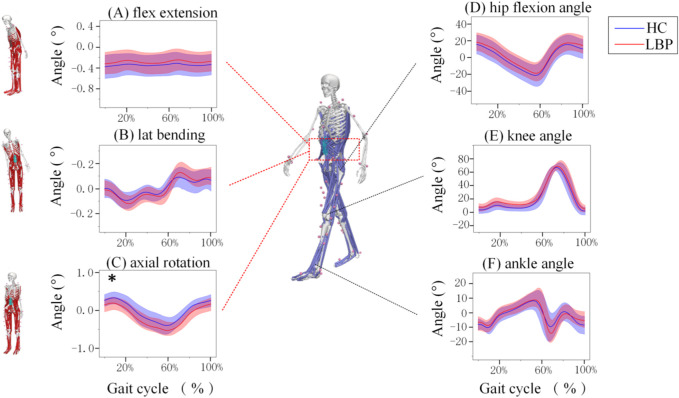
Motions of the joints of the Lumbar and lower limb joints during one gait Cycle. **(A)** The angle of lumbar flexion extension during a gait cycle. **(B)** The angle of lumbar lateral bending during a gait cycle. **(C)** The angle of lumbar axial rotation during a gait cycle. **(D)** The angle of hip motion during a gait cycle. **(E)**The angle of knee motion during a gait cycle. **(F)** The angle of ankle motion during a gait cycle. Lumbar extension is positive, flexion is negative, lateral flexion to the right is positive, lateral flexion to the left is negative, left rotation is positive, and right rotation is negative. The hip joint flexion is positive, extension is negative; knee joint flexion is negative, extension is positive; ankle joint dorsiflexion is positive, plantar flexion is negative. The shaded area represents ±1 standard deviation. *Represents significant differences between HC and LBP groups (*p* < 0.05).

Figure 2 (A, B and C) shows the of lumbar flexion, extension, lateral flexion, and rotation angles during one walking cycle in the two groups. As shown in Figure 2, the changes in lumbar flexion, lateral flexion, and rotation angles during walking in the two groups are consistent. In the LBP group, the lumbar flexion angle was slightly less than that in the healthy group (Figure 2A); the lateral bending angle was slightly greater than that in the healthy group (Figure 2B) and the axial rotation angle was significantly greater than those in the healthy group (Figure 2C).

The changes in hip, knee, and ankle joint angles during walking for the LBP group and the healthy group are shown in Figures 2D–F. The flexion and extension angle changes of the hip, knee, and ankle joints during walking in the LBP group and healthy group are consistent. Throughout the walking process, the flexion angles of the hip and knee joints in the LBP group were greater than those in the healthy group, and the extension angle of the hip joint in the LBP group was less than that in the healthy group; moreover, the peak flexion angles of the hip and knee joints in the LBP group were greater than those in the healthy group, and the peak extension angle of the hip joint in the LBP group was less than that in the healthy group (Figures 2D–F). The peak dorsiflexion angle and plantar flexion angle of the ankle joint in the LBP group were greater than those in the healthy group (Figure 2F; Table 2).

**TABLE 2:** Maximum joint angle (X (sd)).

Characteristic	Low back pain group (n = 19)	Healthy control group (n = 18)	*P*
lumbar flexion and extension angle (°)	−0.23 (0.20)	−0.29 (0.22)	0.375
lumbar lateral bending angle (°)	0.14 (0.37)	0.13 (0.70)	0.396
lumbar axial rotation angle (°)	0.24 (0.18)	0.37 (0.17)	**0.033**
hip flexion angle (°)	20.14 (9.52)	17.32 (11.37)	0.419
knee joint angle (°)	70.06 (4.44)	70.31 (4.88)	0.663
ankle joint angle (°)	17.00 (4.95)	15.94 (6.02)	0.559

The bold values indicate *P* value ≤0.05.

### 3.3 Dynamics

**FIGURE 3:**
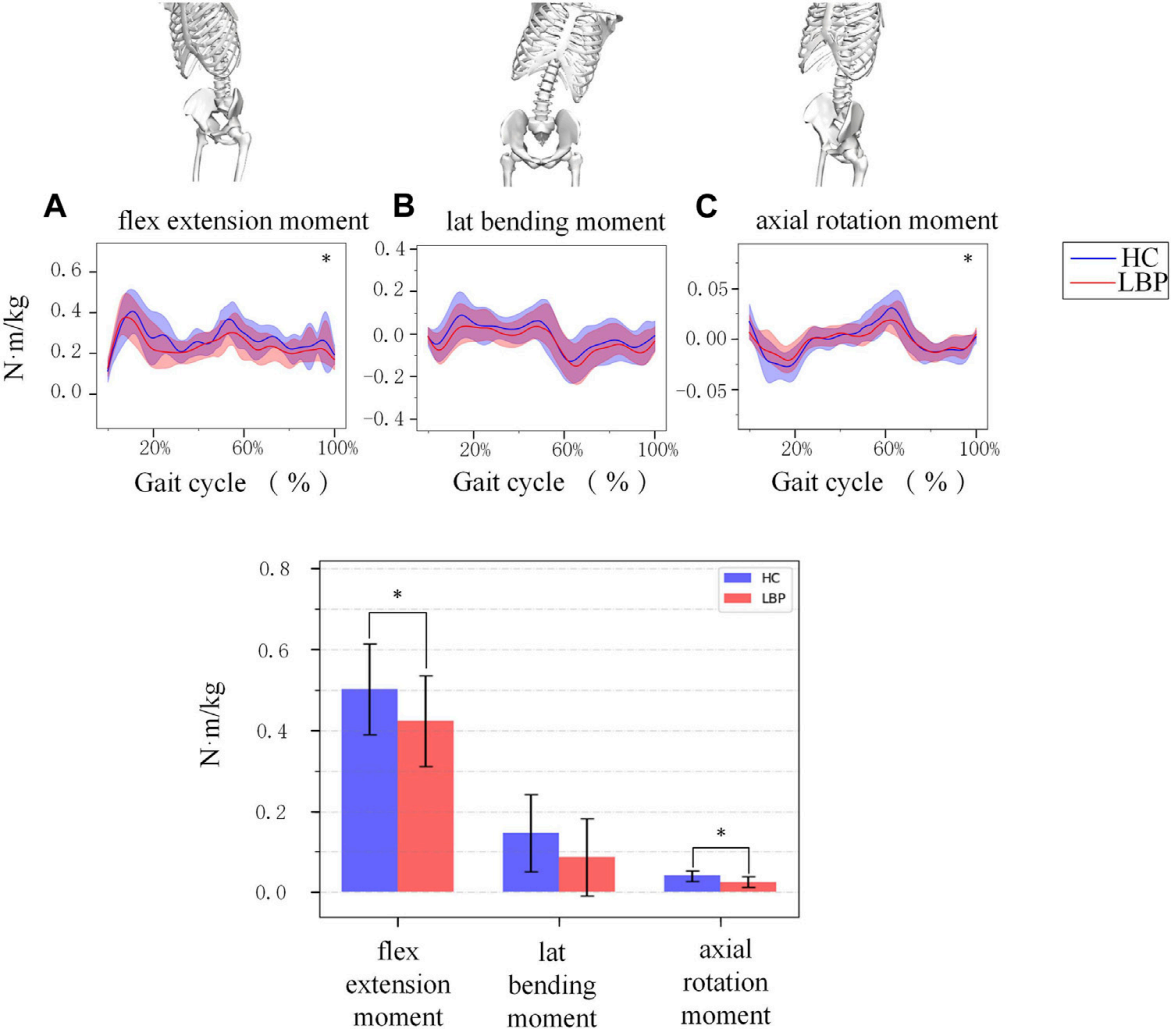
**(A)** The moment of lumbar flexion extension during a gait cycle. **(B)** The moment of lumbar lateral bending during a gait cycle. **(C)** The moment of lumbar axial rotation during a gait cycle. The shaded area represents ±1 standard deviation. The bar chart is a comparison of the moment of the three degrees of freedom of the lumbar spine. *Represents significant differences between HC and LBP groups (*p* < 0.05).

Figure 3 shows the simulation curves of lumbar flexion extension (Figure 3A), lateral flexion (Figure 3B), and rotation (Figure 3C) moment changes during one gait cycle in the two groups. Overall, the changes in lumbar moment during walking in patients with LBP and healthy individuals are relatively consistent. In a standardized gait cycle, the total lumbar extension, right lateral flexion, and right and left rotation moment in the LBP group are less than those in the healthy group. The extension and axial rotation peak moment in the LBP group were significantly lower than those in the healthy group (Table 3, *p* < 0.05), but there was no statistically significant difference in lateral bending peak moment (Table 3, *p* > 0.05).

**TABLE 3:** Maximum joint moment (X (sd)).

Characteristic	Low back pain group (n = 19)	Healthy control group (n = 18)	*P*
lumbar flexion and extension moment (N·m/kg)	0.42 (0.08)	0.50 (0.11)	**0.012**
lumbar lateral bending moment (N·m/kg)	0.09 (0.07)	0.15 (0.10)	0.070
lumbar axial rotation moment (N·m/kg)	0.03 (0.01)	0.04 (0.01)	**0.001**

The bold values indicate *P* value ≤0.05.

### 3.4 Lumbar intervertebral load

**FIGURE 4:**
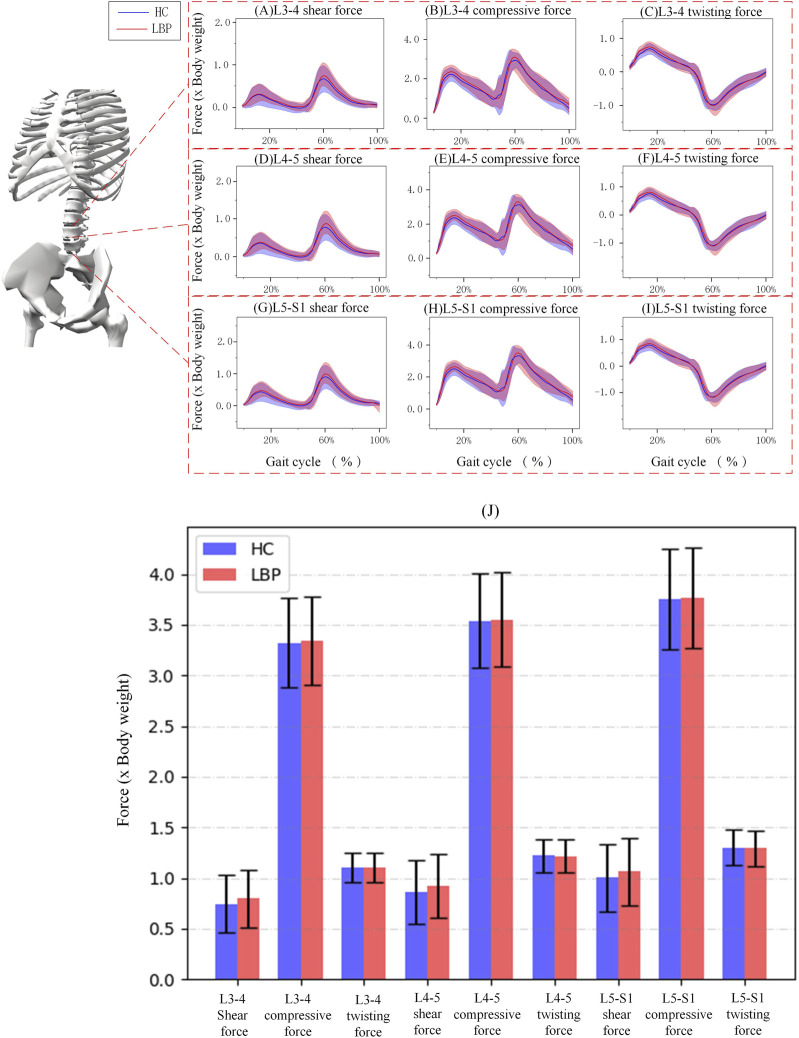
**(A–C)** Corresponding to the shear force, compressive force and twisting force of L3-4 respectively. **(D–F)** Corresponding to the shear force, compressive force and twisting force of L4-5 respectively. **(G–I)** Corresponding to the shear force, compressive force and twisting force of L5-S1 respectively. **(J)** The shaded area represents ±1 standard deviation. The bar chart shows the shear force, compressive force and twisting force of each section of L3-S1.

Figure 4 shows the simulation curves of changes in L3/L4, L4/L5, L5/S1 intervertebral compression force, sagittal shear force, and twisting force during one gait cycle in the two groups. Overall, the intervertebral loads of L3/L4 (Figures 4A–C), L4/L5 (Figures 4D–F), L5/S1 (Figures 4H–J) in patients with LBP are greater than those in healthy individuals. The peak compression force, sagittal shear force, and twisting force of L3/L4, L4/L5, L5/S1 intervertebral discs in patients with LBP are greater than those in healthy individuals, but there is no significant difference (Figure 4K; Table 4, *p* > 0.05).

**TABLE 4:** Maximum lumbar intervertebral force (X (sd)).

Characteristic	Low back pain group (n = 19)	Healthy control group (n = 18)	*P*
L3-4 shear force (x body weight)	0.80 (0.27)	0.74 (0.29)	0.575
L3-4 compressive force (x body weight)	3.34 (0.54)	3.32 (0.45)	0.912
L3-4 twisting force (x body weight)	1.10 (0.22)	1.11 (0.15)	0.965
L4-5 shear force (x body weight)	0.92 (0.29)	0.86 (0.32)	0.560
L4-5 compressive force (x body weight)	3.55 (0.57)	3.54 (0.48)	0.939
L4-5 twisting force (x body weight)	1.22 (0.23)	1.22 (0.17)	0.929
L5-S1 shear force (x body weight)	1.07 (0.32)	1.00 (0.34)	0.555
L5-S1compressive force (x body weight)	3.77 (0.61)	3.76 (0.51)	0.964
L5-S1 twisting force (x body weight)	1.30 (0.24)	1.30 (0.18)	0.913

### 3.5 Muscle force and activition

**FIGURE 5:**
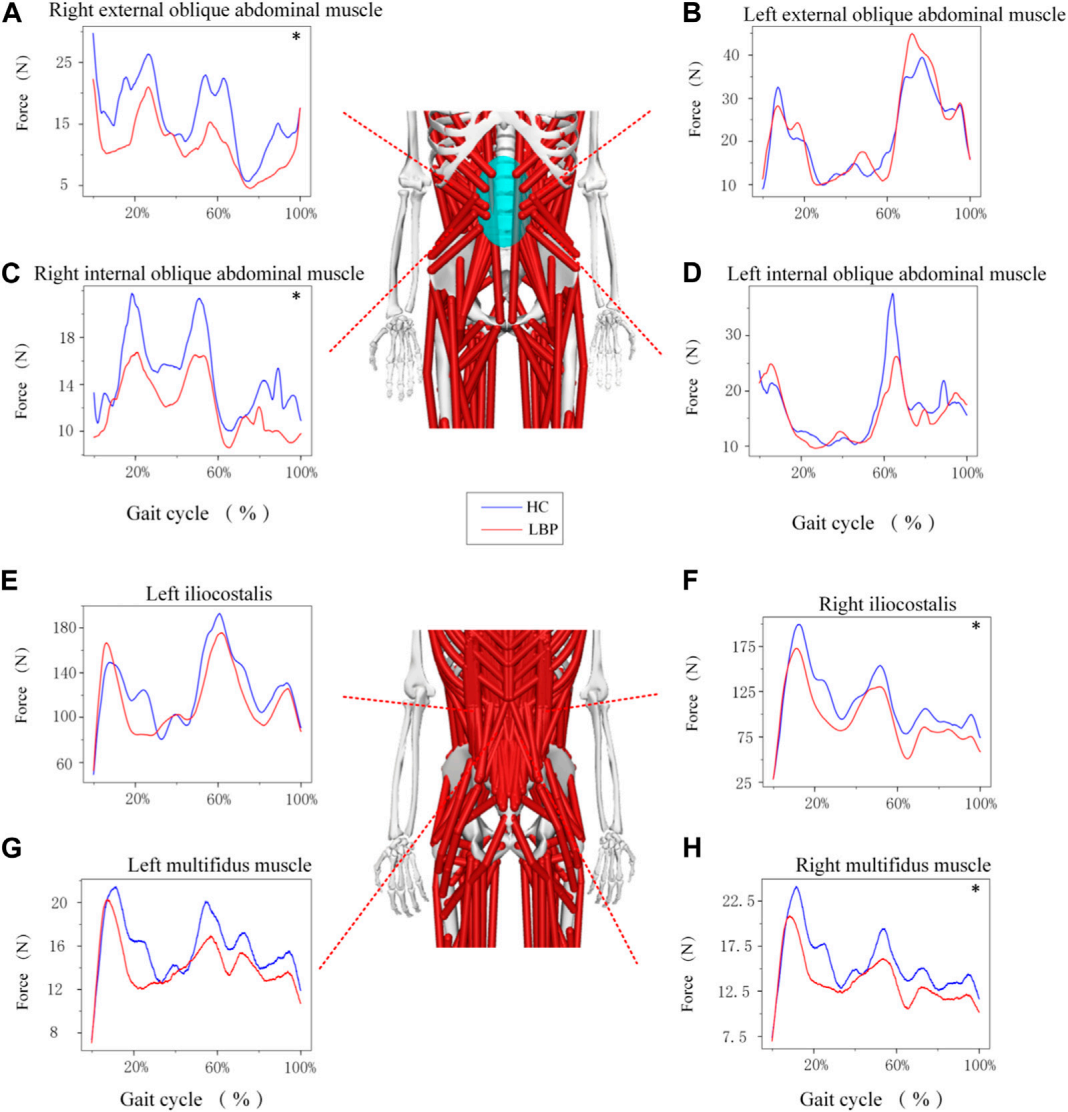
**(A–H)** Represents the force of different muscles during gait. *Represents significant differences between HC and LBP groups (*p* < 0.05).

**FIGURE 6:**
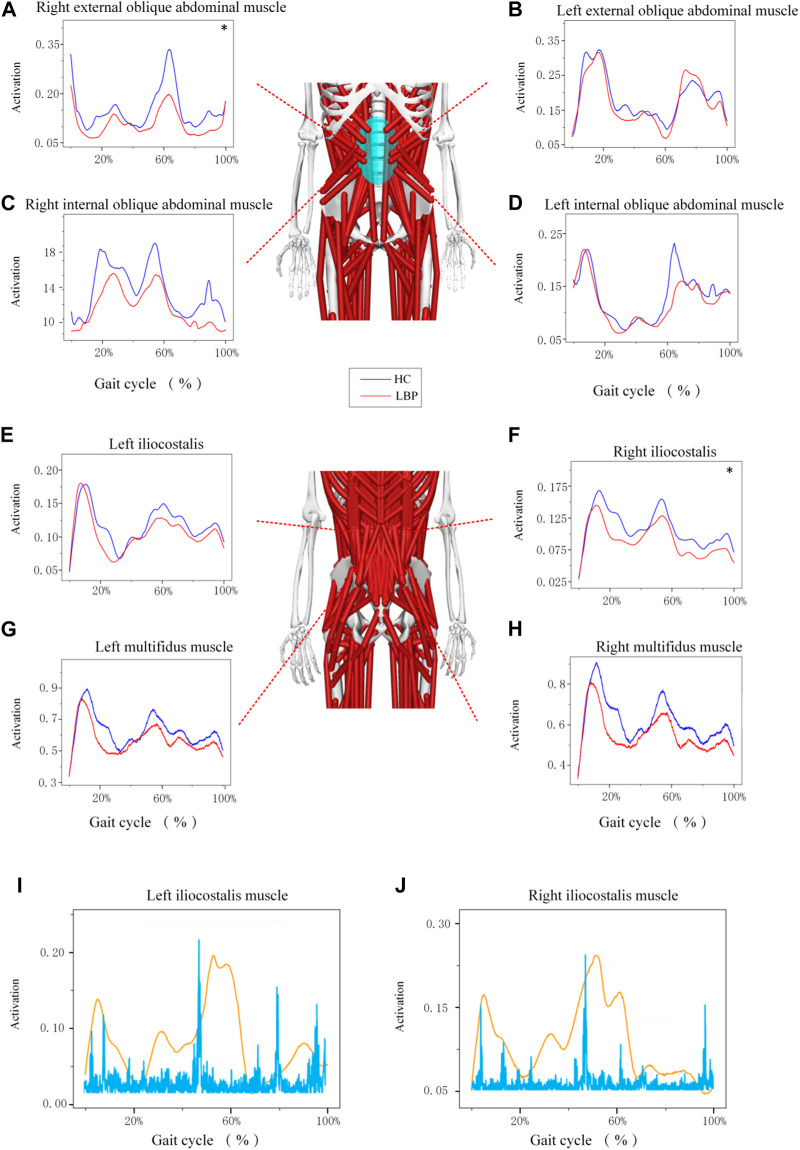
**(A–H)** Represents the activation of different muscles during gait. **(I–J)** Represents a comparison between different muscle activation and EMG data. *Represents significant differences between HC and LBP groups (*p* < 0.05).

The trend of muscle force changes in the LBP group and the healthy group during the gait cycle was similar. The muscle force of the right and left multifidus muscles in the healthy group was higher than that in the LBP group throughout the gait cycle (Figures 5G,H). The muscle force of the right iliocostalis and internal oblique muscles in the healthy group was higher than that in the LBP group throughout the gait cycle (Figures 5C,F), but the muscle force of the left iliocostalis and internal oblique muscles in the first peak was lower than that in the LBP group, and the second peak was higher than that in the LBP group (Figures 5D,H). The muscle force of the right external oblique muscle in the healthy group was higher than that in the LBP group throughout the gait cycle (Figure 5A), but the muscle force of the left external oblique muscle in the first peak was higher than that in the LBP group, and the second peak was lower than that in the LBP group (Figure 5B). There were significant differences in peak muscle force between the two groups for the right multifidus, iliocostalis, internal oblique, and external oblique muscles (*p* < 0.05), but there were no significant differences in peak muscle force for the left multifidus, iliocostalis, internal oblique, and external oblique muscles (Table 5, *p* > 0.05). The simulated muscle activation patterns (Figure 6) were almost consistent with the trend of the above muscle force results, with significantly lower activation of the right external abdominal oblique muscle and right iliocostalis muscle (Table 6, *p* < 0.05) in patients with LBP compared to the healthy control group. In this study, the simulation data of muscle activation in OpenSim was compared with surface EMG signals, and the two showed good consistency (Figures 5I,J).

**TABLE 5:** Maximum muscle force (X (sd)).

Characteristic	Low back pain group (n = 19)	Healthy control group (n = 18)	*P*
right external oblique abdominal muscle(N)	35.11 (11.97)	89.79 (118.46)	**0.014**
left external oblique abdominal muscle(N)	57.73 (20.66)	94.70 (111.23)	0.935
right internal oblique abdominal muscle(N)	25.10 (11.53)	98.56 (190.20)	**0.048**
left internal oblique abdominal muscle(N)	38.35 (17.30)	145.97 (274.54)	0.257
left iliocostalis(N)	220.32 (64.23)	277.19 (151.51)	0.589
right iliocostalis(N)	196.56 (64.41)	259.08 (96.96)	**0.037**
left multifidus muscle(N)	22.70 (7.61)	30.08 (13.62)	0.109
right multifidus muscle(N)	23.11 (8.45)	30.76 (13.34)	**0.048**

The bold values indicate *P* value ≤0.05.

**TABLE 6:** Maximum muscle activation (X (sd)).

Characteristic	Low back pain group (n = 19)	Healthy control group (n = 18)	*P*
right external oblique abdominal muscle	0.33 (0.10)	0.48 (0.20)	**0.004**
left external oblique abdominal muscle	0.42 (0.09)	0.51 (0.22)	0.502
right internal oblique abdominal muscle	0.20 (0.07)	0.29 (0.14)	0.076
left internal oblique abdominal muscle	0.27 (0.10)	0.37 (0.18)	0.125
left iliocostalis	0.20 (0.07)	0.22 (0.09)	0.333
right iliocostalis	0.18 (0.06)	0.22 (0.06)	**0.041**
left multifidus muscle	0.92 (0.31)	1.08 (0.39)	0.151
right multifidus muscle	0.90 (0.31)	1.11 (0.38)	0.082

The bold values indicate *P* value ≤0.05.

## 4 Discussion

This study aims to compare the differences in kinematics, dynamics, lumbar load and muscle force between healthy individuals and patients with chronic LBP using biomechanical methods. The results show significant differences in dynamics and muscle force.

### 4.1 Kinematics

During gait simulation, we compared the lumbar and lower limb joint angle between subjects with LBP and healthy controls. The results indicate that the joint angle in the sagittal of the lumbar spine in subjects with LBP is less than that of healthy subjects, which is consistent with a previous study (Hernandez et al., 2017). Several potential explanations include subjects with LBP limiting their movement to avoid discomfort, stiffness in soft tissues or joints restricting lumbar joint angle, or subjects adopting habitual movement patterns, either the resulting from or contributing to LBP. In the coronal plane, however, lumbar joint angle was greater than that of healthy subjects. This might be explained by lateral instability of the lumbar spine, which also aligns with a previous study suggesting that increased coronal joint angle is related to symptoms in patients with more pronounced degenerative L4-5 spondylolisthesis (Wang et al., 2022). The presence of lateral instability of the lumbar spine during posture changes could lead to more pronounced effects on reported outcomes for patients. Additionally, hip range of motion was different from that of healthy subjects, consistent with the research previous studies (Shum et al., 2005a; Shum et al., 2007; Lee et al., 2011). This may relate to symptomatic subjects employing compensatory movements and altered load-sharing strategies (Ebrahimi et al., 2017; Zawadka et al., 2018)or reduced coordination capabilities of the lumbar spine relative to the hip joint (Shum et al., 2005a). In lower limb joints, the knee joint angle changes were similar between the patient group and healthy subjects, aligning with prior research (Muller et al., 2015; Kuai et al., 2017b). However, the plantar flexion and dorsal extension during mid-stance of the patients with LBP was greater compared to the control group, which might indicate that the altered ankle movement pattern in the sagittal plane might be a compensatory strategy to avoid downward displacement of the center of gravity and reduce mechanical load on the lumbar spine (Zahraee et al., 2014). Furthermore, it is essential to recognize the significant individual differences in human joint motion. This inherent variability may serve as a potential confounding factor in our study.

### 4.2 Dynamics

The lumbar moment of patients with LBP is significantly lower than that of healthy controls in sagittal and axial planes (*p* < 0.05). The change of moment in sagittal plane of lumbar vertebra is consistent with the previous research results of Shum et al. (2005b). Shum’s research results suggest that the activation of the lumbar muscles in patients with LBP, especially the lumbar extensor muscles, is reduced, and the muscle strength is decreased. The moment in sagittal plane of the lumbar is decreased, which may be a compensatory protective mechanism to protect the painful tissues in the waist and back and enhance the stability of the trunk during activity. The decrease of lumbar moment may be related to the “pain-spasm-pain” mode (van Dieen et al., 2003). The occurrence of LBP can cause the co-contraction of the prime mover and antagonist muscles in the waist, and the contraction of the antagonist muscles can lead to the decrease of muscle moment in that area. Although there is no statistically significant difference in the change of moment in coronal plane, we believe that the smaller angle formed by the waist muscle and the coronal plane of the spine may lead to the elongation of the resistance arm (Kalimo et al., 1989; Park et al., 2018), which may be the reason why the change of lumbar sagittal moment is not obvious.

### 4.3 Lumbar intervertebral load

Our results of lumbar compression and shear forces exhibited a typical bimodal pattern, which was closely related to the single-leg standing phase of the left and right feet during the gait cycle (Sung and Danial, 2017). Meanwhile, peak unilateral twisting forces were observed during the single-leg standing phase of each foot, respectively (Paul, 1989). Our study revealed that the peak compressive forces at the L4-L5 segment of the lumbar spine during walking were 3.55 ± 0.57 times and 3.54 ± 0.48 times the body weight for the subjects with LBP and the healthy control group, respectively. This result is generally consistent with the maximum compressive force of 3.45 times the body weight calculated by Callaghan (Callaghan et al., 1999) using a muscle-driven model. The results of lumbar compressive forces obtained by Banks et al. (Banks et al., 2022) using OpenSim for gait simulation were also consistent in terms of trends and magnitudes. There were only slight differences but no significant differences in shear force, compressive force, and twisting force between each lumbar segment in the LBP group and the healthy control group. Nevertheless, we still observed that the shear force, compressive force, and twisting force in the L3-4, L4-5, and L5-S1 segments were slightly greater in patients with LBP compared to the healthy control group. Excessive mechanical loads in daily life may cause structural damage to the intervertebral discs and lead to the occurrence of LBP (Creighton et al., 2023). Similar results were also found in a study by Beaucage-Gauvreau et al. (Beaucage-Gauvreau et al., 2019) comparing patients with LBP and healthy controls under low-load bending conditions. The load on the spine is mainly caused by (1) gravitational forces due to the mass of body segments, (2) external forces and moments induced by a physical activity, and (3) muscle tension (Patwardhan et al., 2008). These loads are distributed among the osseoligamentous tissues and muscles of the spine. Specifically, tensile forces in the paraspinal muscles counterbalance the moments generated by gravitational and external loads, maintaining spinal stability. In this study, there was no statistical difference in body weight between the two groups, and muscle tension was the most likely cause of increased lumbar loads. However, some related studies suggest that significant differences in lumbar loads only occur when muscle contractility is further reduced (Qin et al., 2022). Therefore, we believe that the reason for the absence of significant differences in lumbar loads may be that the subjects with LBP recruited in this study were generally young and their muscle condition could still compensate to temporarily alleviate the excessive mechanical loads on the lumbar spine, compensating for creep and fatigue of the lumbar spine (Shin et al., 2009).

### 4.4 Muscle force and activation

Our results of the peak force of the right multifidus, iliocostalis, and internal and external abdominal oblique muscles in patients suffering with LBP were significantly lower than those of the healthy individuals during walking. This finding is consistent with previous studies which confirmed that a decrease in the activation of the multifidus and iliocostalis muscle in LBP (Danneels et al., 2002; Smith and Kulig, 2016). Our study further highlights the reduction of the right internal and external abdominal oblique muscles force in LBP patients during walking, which echoes the findings in the previous study (Hanada et al., 2011). The pain adaptation theory cannot be ruled out as a possible explanation, which suggests that musculoskeletal pain can lead to a decrease in muscle activity when the muscle as a prime mover (Vogt et al., 2003). Another possible explanation is that pain could lead to muscle changes and compensatory actions from other muscles, or incorrect recruitment patterns in daily activities could result in selective muscle atrophy, inducing pain (Li et al., 2021). Interestingly, in this study, the majority of patients experienced pain on the right side, leading to more pronounced differences in peak muscle force on the right side during walking, while no statistical differences were observed on the left side. This suggests that the adoption of compensatory patterns may have played a significant role. We also observed that although there was a significant difference in the force of the right multifidus muscle between the two groups, there was no significant difference in muscle activation. This may be influenced by muscle thickness, as the healthy control group exhibited significantly thicker multifidus muscle in a contracted state compared to the LBP group. Thicker multifidus muscles can generate more muscle force even under similar activation conditions, contributing to lumbar stability.

### 4.5 Limitations

The study is the first to investigate the loading of the lumbar spine during walking under holistic exercise using experimental data from patients with chronic LBP and healthy controls based on the FBLS model. However, there are some limitations to this study. First, in the simulation of the model, although OpenSim provides many pre-defined mannequins, these models are still simplified approximations and may differ from real human motion situations, for the FBLS model, it is important to note that spinal curvature was not subject-specific, which may affect the calculation of lumbar load. Second, as part of the study exploring the efficacy of remote, smartphone-based interventions for managing LBP which may require complex use of mobile phones and the acceptability of tele-rehabilitation, the recruited subjects were mainly young adults (21–27 years old). Yet, the lumbar structural degeneration is not severe in these young subjects with LBP, which can be compensated for by excessive muscle contraction. Our future work should be to expand the age range of subjects. Finally, in the design of the experiment, this experiment did not conduct subcompositional data on subjects’ LBP location and dominant side. Third, this study only investigated the walking at a comfortable speed, and the lumbar spine load was not measured during fast or slow walking.

## 5 Conclusion

Our study has identified statistical differences in dynamics, muscle force and activation between patients with LBP and healthy controls, indicating that the lumbar moment, muscle force and activation on the affected side are significantly lower in LBP patients. While no statistical difference was found in the loads of the lumbar intervertebral disc, possibly due to the low pain scores in our sample. Future research should consider including a more diverse sample population, with varying age groups and pain levels, to determine the influence of these factors more conclusively. Additionally, exploring the potential effects of limited movement to avoid discomfort, tissue stiffness, and habitual movement patterns on lumbar joint angle and muscle force could yield insights into the mechanisms underlying LBP. The results of this study also suggest that the reduction of muscle force and activation on the painful side of the lumbar may play an important role in the occurrence of chronic LBP. In rehabilitation, more attention should be paid to the training of the lumbar muscles, such as core training, with the goal of improving the force and activation of the core muscles, which may achieve good therapeutic effects.

## Data Availability

The raw data supporting the conclusions of this article will be made available by the authors, without undue reservation.
